# 2331. Characteristics Associated with Hospital-Acquired COVID-19 Infections in Los Angeles County

**DOI:** 10.1093/ofid/ofad500.1953

**Published:** 2023-11-27

**Authors:** Heidi D Sato, Kelsey OYong, Zachary Rubin

**Affiliations:** Los Angeles County Department of Public Health, Los Angeles, California; Los Angeles County Department of Public Health, Los Angeles, California; Los Angeles County Department of Public Health, Los Angeles, California

## Abstract

**Background:**

Few studies have characterized nosocomial transmission of SARS-CoV-2. We examined factors associated with hospital-acquired infections (HAIs) rates for SARS-CoV-2 in Los Angeles County (LAC).

**Methods:**

In this retrospective cohort study, data were collected from 70 acute care hospitals between July 1, 2020 and March 31, 2022. We defined COVID HAIs as a patient with laboratory-confirmed SARS-CoV-2 (PCR or antigen test), which was first positive 4 or more days after hospital admission and before discharge. Community transmission (CT) level was calculated by dividing new cases within the past 7 days by the population in the county (per 100,000). Variant predominant periods were defined as periods when the variant exceeded 50% of sequenced specimens during the specimen collection month. The percent of core healthcare personnel (HCP) receiving a completed COVID vaccination series (as of 11/28/2021) was obtained from the National Healthcare Safety Network (NHSN). Incidence rates were calculated using patient-days as a denominator, as reported to NHSN. Mantel-Haenszel test was performed to assess COVID HAIs and predominant variant periods, controlling for effect of CT level. We analyzed hospital characteristics associated with incidence rate using multiple regression (SAS 9.4).

**Results:**

During the 21-month study period, 2107 patients were identified as having COVID HAIs at 70 hospitals using the standardized definition. COVID HAIs were more likely to occur during high CT levels (RR=6.2; 95% CI=4.9, 8.0) and when the Omicron variant was predominant (RR_CMH_ = 1.2; 95% CI=1.1, 1.4). In multivariate analysis, a higher rate of COVID HAIs was associated with hospitals unaffiliated with medical schools (β= -30.9, p< .05), lower HCP vaccination coverage (β= -0.6, p< .05), more hospital beds (β= 0.1, p< .05), and ownership type (β= 19.2, p< .01). In comparison to government hospitals, HAIs were three times more likely to occur in for-profit hospitals (95% CI=2.6, 3.6) and 1.4 times more likely to occur in not-for-profit hospitals (95% CI=1.2, 1.7).

**Conclusion:**

COVID HAIs are more likely to occur during high transmission periods and at hospitals without medical schools, with for-profit ownership, lower HCP vaccination rates, and with a higher number of beds.

**Disclosures:**

**All Authors**: No reported disclosures

